# Adverse health outcomes in testis cancer survivors—the price for our success?

**DOI:** 10.1093/oncolo/oyaf117

**Published:** 2025-06-14

**Authors:** Danish Mazhar, Sophie Heritage, Han Wong

**Affiliations:** Department of Oncology, Cambridge University Hospitals NHS Foundation Trust, Cambridge, Cambs CB2 0QQ, UK; Department of Medicine, Cambridge University Hospitals NHS Foundation Trust, Cambridge, Cambs CB2 0QQ, UK; Department of Oncology, Cambridge University Hospitals NHS Foundation Trust, Cambridge, Cambs CB2 0QQ, UK

Testicular cancer (TC) is the most common malignancy in men aged less than 35 years of age, with approximately 90% of the patients being less than 55 years. Approximately 1:200 males in the UK develop TC in their lifetime. Although the worldwide incidence of TC is low, it is estimated to have doubled in the past 40 years and there is appreciable variation between countries. The highest rates of TCs are reported for white Caucasian populations in industrialized countries, particularly in Western and Northern Europe and Australia/New Zealand.^1^

Testicular germ cell tumors, which make up the vast majority of TCs, are highly chemosensitive. However, until a few decades ago, deaths following a diagnosis of metastatic disease were common. Some patients with metastatic disease were successfully treated by radiotherapy, but outcomes were poor with only 5%-10% of patients cured. In the 1970s there were significant advances in therapies. The introduction of cisplatin-containing chemotherapy for the treatment of metastatic germ cell tumors by the Indiana group in the late 1970s has to be considered as one of the single most important advances in oncology.^2,3^ Across all stages, the 10-year survival rate of TC is now approximately 95%, and the cure rate for patients with stage 1 disease approaches 100%.

While the modern management of TC undoubtedly represents a medical success story, the impact of treatment-related complications in TC survivors has become an increasing concern in recent years. Platinum, a crucial component of the chemotherapeutic management of TC, remains partially active and measurable over a decade after treatment and there are short, medium, and long-term impacts of radiotherapy on exposed surrounding normal tissue and on organ function.

The treatment of TC with cisplatin-containing chemotherapy is associated with a wide range of potential toxicities including myelosuppression, infection risk (including febrile neutropenia), emesis, tinnitus, high-tone hearing loss, nephrotoxicity, neuropathy, and alopecia. Advances in supportive medications such as growth factors, 5-HT3 antagonists, and neurokinin inhibitors have helped to reduce the risk and impact of some serious short-term adverse effects, but the treatment remains significantly toxic and hard to tolerate for many patients.

Bleomycin, given as part of the BEP (bleomycin, etoposide, cisplatin) regimen, can cause lung toxicity, which can potentially be fatal. Bleomycin lung toxicity is considered dose-related, though there are risk factors such as age and poor renal function that can elevate risk. It is characterized by pneumonitis in the initial stages, and this can then be followed by progressive pulmonary fibrosis which has a mortality of 1%-2%.

Long-term side effects of platinum-containing chemotherapy include a raised body mass index, and an elevated risk of hypertension, hyperlipidemia, and cardiovascular disease. One study reported treatment with BEP increased the risk of coronary artery disease by 5.7-fold (95% CI, 1.9-17.1) compared with surgery only, while the risk for myocardial infarction increased by 3.1-fold (95% CI, 1.2-7.7) compared with age-matched male controls.^4^ There is an increase in secondary malignancies in TC survivors treated with platinum-based chemotherapy, occurring with a relative risk (RR) of 1.4 to 2.4.^5-8^ Radiotherapy can also induce an increased risk of second cancers. A large study of over 40 000 testis cancer survivors reported a 2.7-fold increased risk of solid cancers within the infradiaphragmatic radiotherapy fields.^9^

Other significant physical health issues identified in testis cancer survivors include hypogonadism and infertility. A meta-analysis by Bandak et al^10^ reported a higher risk of hypogonadism with both standard cisplatin-based chemotherapy (RR 1.8) and infradiaphragmatic radiotherapy (RR 1.6). Hypogonadism has been associated with reduced sexual and social functioning, fertility problems, chronic fatigue, osteoporosis, metabolic syndrome, and cardiovascular disease. Many TC survivors also experience mental health issues such as anxiety and depression. One review highlighted that TC survivors experience more clinically significant anxiety than the general population,^11^ and studies have reported depression rates approaching 10% in TC survivors^12^ with an elevated risk of suicide also reported.^13,14^

Whilst the long-term physical and psychosocial morbidity associated with TC treatment is clearly an area of concern, there is now compelling evidence that TC survivors have heightened mortality risks. Hellesness et al^14^ investigated the impact of TC management on non-testis cancer mortality, and reported a 23% excess in mortality compared with the general population with significantly elevated risks after platinum-based chemotherapy but not after surgery. The non-testis cancer mortality was highest among survivors diagnosed under the age of 20 years who had a 2.27-fold increased risk. Consistent with these data, a Dutch study reported a 40% significant excess of non-testis cancer mortality among over 6000 TC survivors compared with the general population, with a cumulative mortality of 9.6% 25 years after treatment.^15^

Second malignancies were found by Hellesness et al to be the most important cause of non-testis cancer mortality in TC survivors (standardized mortality ratio of 1.53) with 43% and 59% excess deaths after platinum-based chemotherapy and infradiaphragmatic radiotherapy respectively,^14^ Both 4 and >4 cycles of cisplatin-containing chemotherapy were associated with excess deaths on multivariable analyses. Groot et al also demonstrated a dose relationship between cisplatin exposure and the risk of second malignancies.^15^

There is increased mortality from cardiovascular disease in patients treated for TC. Fossa et al^16^ found a 60% excess mortality from circulatory disease among TC survivors given chemotherapy after 1975, and Groot et al reported a 2.1-fold risk of cardiac mortality after platinum-based chemotherapy compared to patients not exposed to platinum.^15^

A North American study found that approximately one-third of TC survivors have at least 3 adverse health outcomes (AHOs) following platinum-based chemotherapy.^17^ The cumulative burden of morbidity (CBM) was examined by a further study that evaluated both the number of AHOs and their severity. This found that around 20% of TC survivors had a CBM score of high, very high, or severe, with close to 30% scoring medium.^18^

Whilst TC cure rates are highly favorable, there remain areas where improvements in patient outcomes are needed, such as adverse risk metastatic disease and relapse following treatment for metastatic cancer. Despite the clear burden of toxicity associated with treatment, current studies are focusing on therapy intensification in these disease settings. Whilst this is perhaps understandable, there has to be a concern regarding the long-term health impacts of such approaches. We believe the development of more individualized therapeutic algorithms based on better risk stratification through the identification of novel biomarkers should be a key aim. Recent reports of microRNAs as potential biomarkers in TC represent an important development in this regard.^19^ Such research will be crucial in enabling the de-escalation of therapy in patients with more favorable disease parameters and in the development of a more comprehensive assessment of risk in patients with stage 1 disease. Epigenetic research is also crucial in the identification of further variants that predispose to treatment-related toxicities, such as the germline mutations that have been implicated in platinum-related ototoxicity and neuropathy.

Clinicians and patients are understandably concerned about relapse after treatment of TC. For patients on active surveillance after orchidectomy for stage 1 disease, early identification of disease relapse is important. For patients treated for metastatic disease (and indeed in stage 1 TC patients as well) the reality is that most patients are cured after first-line treatment and relapse is relatively rare beyond 2 years of treatment completion. And yet, our management of these patients is still largely focused on identifying disease progression, and whilst there has been an increasing appreciation of the need to screen for late treatment effects, this may be a secondary concern for clinicians and indeed patients. There is a risk that this may be even more of an issue in the future with the increasing utilization of virtual consultations and patient-directed follow-up. We believe there needs to be a fundamental shift in our approach to the follow-up of TC survivors. We need to move from being passive and reactive to health issues as they arise to fostering a more proactive strategy with an increased emphasis on long-term health and its outcomes, and a greater focus on reducing the risk of known associated chronic diseases. The question of who is best placed to manage patients with TC-treatment-associated toxicities should also be raised. The medical needs of TC survivors may potentially be complex, and whilst Oncologists and Cancer Nurse Specialists should play a leading role in the screening for treatment-related toxicities, optimal clinical management will involve other specialties including primary care, endocrinology, cardiology, and psychiatry. Lifestyle changes should be promoted during TC follow-up, and we support, as has been proposed, the adoption of a cancer survivorship plan addressing lifestyle changes and recommendations and late toxic effects.^20^ The favorable oncological outcomes of TC treatment should rightly be applauded, but we need to be mindful of the burden of treatment-related toxicity and do more to improve the lives of TC survivors, and not let them pay the price for our apparent success.

## Data Availability

No new data were generated or analyzed for this editorial
